# The Cell Cycle Checkpoint System MAST(L)-ENSA/ARPP19-PP2A is Targeted by cAMP/PKA and cGMP/PKG in Anucleate Human Platelets

**DOI:** 10.3390/cells9020472

**Published:** 2020-02-18

**Authors:** Elena J. Kumm, Oliver Pagel, Stepan Gambaryan, Ulrich Walter, René P. Zahedi, Albert Smolenski, Kerstin Jurk

**Affiliations:** 1 Center for Thrombosis and Hemostasis (CTH), University Medical Center of the Johannes Gutenberg-University Mainz, 55131 Mainz, Germany; elena.kumm@unimedizin-mainz.de (E.J.K.); s.gambaryan@klin-biochem.uni-wuerzburg.de (S.G.); ulrich.walter@uni-mainz.de (U.W.); 2 Leibniz-Institut für Analytische Wissenschaften—ISAS—e.V., 44227 Dortmund, Germany; oliver.pagel@isas.de (O.P.); Rene.Zahedi@ladydavis.ca (R.P.Z.); 3 Sechenov Institute of Evolutionary Physiology and Biochemistry, Russian Academy of Sciences, St. Petersburg 194223, Russia; 4 Proteomics Centre, Lady Davis Institute, Jewish General Hospital, Montréal, QC H3T1E2, Canada; 5 UCD Conway Institute, UCD School of Medicine and Medical Science, University College Dublin, D04 V1W8 Dublin, Ireland; albert.smolenski@ucd.ie

**Keywords:** platelets, serine/threonine protein phosphatases, cyclic AMP, cyclic GMP, ENSA, ARPP19, MAP kinase

## Abstract

The cell cycle is controlled by microtubule-associated serine/threonine kinase-like (MASTL), which phosphorylates the cAMP-regulated phosphoproteins 19 (ARPP19) at S62 and 19e/α-endosulfine (ENSA) at S67and converts them into protein phosphatase 2A (PP2A) inhibitors. Based on initial proteomic data, we hypothesized that the MASTL-ENSA/ARPP19-PP2A pathway, unknown until now in platelets, is regulated and functional in these anucleate cells. We detected ENSA, ARPP19 and various PP2A subunits (including seven different PP2A B-subunits) in proteomic studies of human platelets. ENSA-S109/ARPP19–S104 were efficiently phosphorylated in platelets treated with cAMP- (iloprost) and cGMP-elevating (NO donors/riociguat) agents. ENSA-S67/ARPP19-S62 phosphorylations increased following PP2A inhibition by okadaic acid (OA) in intact and lysed platelets indicating the presence of MASTL or a related protein kinase in human platelets. These data were validated with recombinant ENSA/ARPP19 and phospho-mutants using recombinant MASTL, protein kinase A and G. Both ARPP19 phosphorylation sites S62/S104 were dephosphorylated by platelet PP2A, but only S62-phosphorylated ARPP19 acted as PP2A inhibitor. Low-dose OA treatment of platelets caused PP2A inhibition, diminished thrombin-stimulated platelet aggregation and increased phosphorylation of distinct sites of VASP, Akt, p38 and ERK1/2 MAP kinases. In summary, our data establish the entire MASTL(like)–ENSA/ARPP19–PP2A pathway in human platelets and important interactions with the PKA, MAPK and PI3K/Akt systems.

## 1. Introduction

Platelets are small, anucleate blood cells, which are essential in physiological and pathological haemostasis but also have important roles in inflammation, atherosclerosis and cancer [1,2,3,4,5]. In the process of haemostasis, platelets are regulated by multiple endogenous activating and inhibitory factors that normally prevent spontaneous platelet adhesion to the vessel wall and subsequent platelet aggregation, thrombus formation and occlusion of blood vessels. Upon vascular injury, platelets adhere to the injured endothelium and to subendothelial matrix proteins such as collagen to form localized thrombi and prevent blood loss. Either inherited or acquired dysfunctions of human platelets may cause serious, even lethal bleeding or thrombotic complications [3,6]. In addition to their essential regulation of haemostasis and coagulation, activated platelets release and secrete, primarily from their α- and δ-granules, more than 300 biomolecules and proteins, which affect other platelets, blood, vascular and tissue cells [7,8]. These secreted platelet factors regulate multiple physiological and pathophysiological processes such as microvascular integrity, wound healing, inflammation, tumour stability and metastasis [7,8]. Among the important factors released are thromboxane A2 (TXA2) and adenosine 5’-diphosphate (ADP), which enhance the initial platelet response and recruit additional platelets to the growing thrombus. Targeting and blocking of enhanced platelet activation by TXA2 synthesis inhibitors (aspirin) and/or ADP-receptor antagonists (thienopyridines) have been well established as the most effective intervention to prevent and attenuate complications of various acute and chronic cardiovascular diseases [9].

In vivo, platelets are activated by subendothelial collagen predominantly via their cell membrane GPVI/FcRγ-chain receptor complex. Other platelet agonists such as von Willebrand factor (vWF) and podoplanin bind to and stimulate specific platelet membrane-receptor complexes such as GPIb/V/IX and the C-type lectin receptor CLEC-2, respectively. Soluble agonists such as thrombin, ADP and TXA2 bind to and activate G-protein coupled receptors (GPCRs). The resulting platelet activation is a multistep process, characterized by cytoskeletal rearrangements, integrin activation, granule secretion, TXA2 synthesis/release, and exposure of anionic phospholipids, leading to platelet shape change, adhesion, aggregation and platelet-dependent coagulation [5,10,11]. Platelet activation responses are mediated by tyrosine protein kinases [11], Ca^2+^/calmodulin-dependent protein kinases protein kinase C (PKC) [12,13,14] and phospholipases. On the other hand, endothelial cell-derived prostacyclin and nitric oxide (NO) represent the two major endogenous platelet inhibitors, which increase the level of platelet cAMP and cGMP, respectively [15,16,17]. Elevated cAMP and cGMP levels regulate specific effector systems in platelets such as certain phosphodiesterases (PDEs) and cAMP- and cGMP-dependent protein kinases A (PKA) and G (PKG), which phosphorylate multiple substrates involved in platelet inhibition [18,19]. Overall, responses of both platelet agonists and inhibitors are mediated by an extensive membrane receptor-activated intracellular signalling network that includes intracellular serine/threonine protein kinases/phosphatases and tyrosine protein kinases/phosphatases [10,11,20,21].

Many serine/threonine protein kinases, tyrosine protein kinases and tyrosine protein phosphatases have been extensively studied also in platelets [11,14,22], but much less is known about platelet serine/threonine protein phosphatases and in particular PP2A. This is perhaps due to the substantial heterogeneity and complexity of PP2A. In addition, for a long time serine/threonine protein phosphatases were considered unregulated enzymes, which ‘simply’ limit protein kinase action. However, PP2A is now known to be a heterotrimeric enzyme with more than 90 different forms in humans, playing important roles in cell growth and signalling [23,24,25,26]. PP2A is expressed in most cells from yeast to man and exists in the cell predominantly as a heterotrimeric holoenzyme composed of a catalytic subunit (C), a scaffold subunit (A) and a targeting/regulatory subunit (B) [25,27]. In humans, A- and C-subunits of PP2A each have two possible variants (α, β) whereas the B-subunits are encoded by 15 different genes, which yields at least 23 isoforms due to alternative promoters and alternative splicing. Based on this, human tissues/cells are expected to contain up to 92 different trimeric PP2A holoenzymes and additional four dimeric (AC) forms [25]. Overall, the B-type subunits are true ‘regulatory’ subunits, which determine substrate specificity of the associated PP2A C-subunit and modulate PP2A catalytic activity. They are often expressed in a specific way and determine the intracellular location of the PP2A holoenzymes. 

The cAMP-regulated phosphoproteins (ARPP) with ENSA (also known as ARPP19e or α-endosulfine), ARPP19 and its isoform ARPP16 were discovered and studied extensively as PKA substrates in cells other than platelets, with limited functional information [28]. However, considerable progress was made in 2010 when two independent groups reported for *Xenopus* oocytes that both ENSA and ARPP19 inhibit PP2A (only B55δ-subunit) and thereby control mitosis, when phosphorylated by a special kinase called the Greatwall kinase (Gwl) [29,30]. Whereas PKA phosphorylated ENSA/ARPP19 at their C-terminal site S109/S104 with unknown effect, ENSA/ARPP19 phosphorylation at S67/S62 by Gwl was required for the potent inhibition of PP2A [31]. ENSA and ARPP19 are highly conserved [especially the central region with the Gwl phosphorylation site] in a broad spectrum of systems such as plants, *Xenopus*, *Drosophila*, and a wide range of eukaryotes including yeast and humans [32]. Since the original demonstration in 2010, multiple studies established that the Gwl/MASTL-ENSA/ARPP19–PP2A pathway is an important check-point for controlling mitosis and its M- and S-phases from yeast to man [33]. However, ENSA/ARPP19 and their targets were not investigated in anucleate platelets until now. In our own proteomic/phosphoproteomic studies with human platelets we discovered both ENSA, ARPP19 [34,35] and in this study other PP2A components. Although possible platelet functions of PP2A were investigated in early studies using global PP2A inhibitors [36,37,38,39], the PP2A composition in human platelets and the regulation and role of ENSA/ARPP19 have not been addressed so far. In the light of the possible, but unknown major regulatory role of the PP2A system as opponent of serine/threonine protein kinases in human platelets, we developed the hypothesis that a MASTL-ENSA/ARPP19-PP2A pathway is present and regulated in human platelets, with important interactions with the cAMP/PKA system. Therefore, we investigated the spectrum of platelet protein kinases that phosphorylate/regulate ARPP19/ENSA, and searched for a Gwl/MASTL-like protein kinase activity, which phosphorylates ARPP19/ENSA and affects serine/threonine protein phosphatases of the PP2A family. Finally, we asked whether the ENSA/ARPP19/PP2A pathway regulates the phosphorylation state of important signaling proteins in platelets. 

## 2. Materials and Methods

### 2.1. Materials

Recombinant GST-ARPP19 and active MASTL kinase were from SignalChem (Richmond, BC V6V 2J2, Canada); DNA for ENSA production, C-subunit of PKA (bovine) and PKGIβ (human) were kindly provided by Prof. E. Butt, University of Würzburg, Würzburg, Germany; 8-Bromo-cGMP sodium salt and fostriecin sodium salt were from Cayman chemical, USA; okadaic acid ammonium salt (OA) was from Enzo Life Sciences, Lörrach, Germany; tautomycetin was from Tocris/Bio-Techne GmbH, Wiesbaden, Germany bovine serum-albumin fraction V (BSA) was from Capricorn Scientific GmbH, Ebsdorfergrund, Germany; isopropyl β-D-1-thiogalactopyranoside (IPTG) and Zeba^TM^ spin desalting columns (0.5 mL, 7K MWCO) were from Thermo Fisher Scientific, Waltham, MA USA; phostag^TM^ AAL-107 was from Wako Chemicals, Neuss, Germany; Clarity^TM^ Western ECL substrate, HRP-conjugated anti-rabbit antibody were from BioRad Laboratories, Hercules, CA, USA; primary antibodies (ENSA pS67/ARPP19 pS62; ENSA general; AKT T308/S473; VASP pS239; p38 MAP kinase pT180/pY182 (12F8); p44/42 MAPK (ERK1/2) pT202/Y204 and α-actinin) were from Cell Signaling Technology^®^, Danvers, MA USA; anti-ARPP19 antibody (rabbit polyclonal serum), anti-PP2A B56δ and anti-PP2A B56δ pS573 antibodies (both rabbit) were kindly provided by Prof. A. Nairn, Yale University, New Haven, CT USA; anti-ENSA pS109 antibody (rabbit) was kindly provided by Prof. Satoru Mochida (Kumamoto University, Kurokami, Chuo Ward, Japan); PAN VASP antibody and antibody against ENSA pS109/ARPP19 pS104 were custom prepared by ImmunoGlobe^®^, Himmelstadt, Germany; HisTrap columns (1 mL) and PD-10 desalting columns were from GE Healthcare, Chicago, IL USA; *E. coli* BL21 (DE3) were from New England Biolabs (NEB), Frankfurt am Main, Germany; pET28a vector was from Novagen/Merck KGaA, Darmstadt, Germany; human embryonic kidney cells 293 (HEK293 cells) were kindly provided by the clinic for obstetrics and women’s health (University Medical Center of the Johannes Gutenberg-University Mainz, Mainz, Germany); HEK293 growth medium (Dulbecco’s Modified Eagle Medium/DMEM) was from Life Technologies Inc./Thermo Fisher Scientific; pCMV-3Tag-1A vector for FLAG-ENSA was from Agilent Technologies, Santa Clara, CA USA; PolyJet^TM^ Transfection reagent was from SignaGen^®^ Laboratories, Rockville, MD USA; Ser/Thr phosphatase activity assay for quantification of PP2A activity was from Promega Corporation, Madison, WI USA; cOmplete^TM^ protease inhibitor mini, thioATP (adenosine 5′-[3-γ-thio]triphosphate) lithium salt, α-thrombin/factor IIa (from human plasma) were from Roche Diagnostics International AG, Rotkreuz, Switzerland; ATP (adenosine 5′-triphosphate) and forskolin were from Sigma-Aldrich GmbH/Merck KGaA. 

### 2.2. Canonical Sequence of ENSA and ARPP19

**Figure 1:**
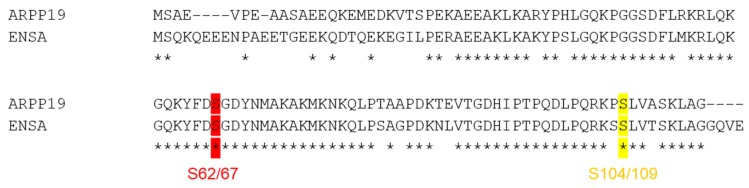
Amino Acid sequence alignment of ENSA isoform 1 and ARPP19. Clustal Omega (Version 1.2.1) was used for sequence alignment. S62/67 is marked in red, S104/109 in yellow. Stars demonstrate identical amino acids. Empty space shows that there is no amino acid sequence similarity between ARPP19 and ENSA.

In the following (Figure 1), the canonical sequences of the ENSA and ARPP19 proteins used for this study (without tags) are shown in comparison. Stars show identity of amino acids. Sequence alignment was performed with Clustal Omega (Version 1.2.1).

### 2.3. Recombinant Wildtype and Mutant ENSA Protein Expression and Purification

*E. coli* BL21 were transfected with pET28 vectors including the DNA for wildtype or mutant (S67A/S109A/S109D) HisENSA. Protein expression was induced with isopropyl β-d-1-thiogalactopyranoside (IPTG) (0.1 mM) and proteins were isolated 20 h after induction. Therefore, *E. coli* were pelleted at 4225× *g* for 10 min at 4 °C. The pellets were resuspended in ice-cold lysis buffer (50 mM NaH_2_PO_4_, 300 mM NaCl, 1 mM MgCl_2_, 15 mM imidazole, pH 8.0, cOmplete^TM^ protease inhibitor cocktail) and four times sonicated with 30 s of resting on ice in between. The lysate was centrifuged for 45 min at 16,900× *g* and 4 °C. The recombinant wildtype and mutant HisENSA proteins were purified using metal ion affinity chromatography (1 mL HisTrap HP (Ni^2+^-ions) columns and an ÄKTA prime, GE Healthcare). The proteins were eluted with elution buffer (20 mM Na_3_PO_4_, 500 mM NaCl, 500 mM imidazole, pH 7.4), afterwards, the buffer was exchanged to 1× TBS buffer (1.37 M NaCl, 0.2 M tris, pH 7.4) with PD-10 desalting columns (GE Healthcare).

### 2.4. Culture, Treatment, and Sample Generation of HEK293 Cells

HEK293 cells were grown in DMEM at 37 °C and 5% (*v*/*v*) CO_2_ on 6-well plates until they reached 80% confluence. They were transfected with pCMV-3Tag-1A vector including FLAG-ENSA sequence using PolyJet^TM^ transfection reagent (1 µg of vector/well). One day after transfection, cells were incubated in the presence or absence of 10 µM forskolin or vehicle control (0.1% (*v*/*v*) DMSO) or iloprost (1 µM) for 15 min or in the presence or absence of thrombin (0.1 U/mL) for 10 min at 37 °C and 5% (*v*/*v*) CO_2_. As control, non-transfected cells were used. Cells were lysed with 2× lysis buffer (0.1 M tris, 0.3 M NaCl, 10 mM MgCl_2_, 2% (*v*/*v*) triton, pH 7.5) and western blot samples were generated in 3× Laemmli buffer, heated for 10 min at 95 °C.

### 2.5. Preparation of Washed Human Platelets

Washed human platelets were isolated from citrate-anticoagulated (3.2% (*v*/*v*) tri-sodium-citrate) whole blood, taken from healthy volunteers not on platelet-affecting drugs for at least 10 days before blood collection. All subjects gave their informed consent for inclusion before they participated in the study. The study was conducted in accordance with the Declaration of Helsinki, and the protocol was approved by the local Ethics Committee of the University Medical Center Mainz (Study No. 837.302.12; 25.07.12; FF109/2015). Platelet-rich plasma (PRP) was generated by centrifugation of citrated blood, supplemented with 2 mM EGTA, at 200× *g* for 10 min at room temperature (RT). PRP was diluted 1:1 with CGS buffer (120 mM NaCl, 12.9 mM trisodium citrate dihydrate, 30 mM d-glucose, pH 6.5) and centrifuged at 69× *g* for 10 min at RT, to pellet the leukocytes. The supernatant was centrifuged at 400× *g* for 10 min at RT. The platelet pellet was resuspended in 3 mL CGS buffer and centrifuged again at 400× *g* for 10 min at RT. Finally, platelets were resuspended in HEPES buffer (150 mM NaCl, 5 mM KCl, 1 mM MgCl_2_, 10 mM d-glucose, 10 mM HEPES, pH 7.4). The isolated platelets were adjusted to 5 × 10^8^ or 1 × 10^9^ or 2 × 10^9^ platelets/mL and kept at 37 °C for 15 min.

### 2.6. Generation of Platelet Lysates from Washed Human Platelets

Washed human platelets were incubated with 1 mM Ca^2+^ in the presence or absence of 2 µM okadaic acid (OA) for 15 min at 37 °C. Platelets were centrifuged at 16,900× *g* for 1 min at 4 °C. The pellet was resuspended in 50% (*v*/*v*) of the original volume of lysis buffer (50 mM tris, 150 mM NaCl, 1 mM EDTA, 0.1 mM EGTA, 0.25% (*v*/*v*) NP-40, pH 7.4; cOmplete^TM^ protease inhibitor cocktail) and vortexed.

### 2.7. Western Blot Analysis

Western blot samples were prepared with 3 x Laemmli buffer (200 mM tris/HCl, 15% (*v*/*v*) glycerol, 6% (*w*/*v*) SDS, 0.06% (*w*/*v*) bromphenol blue; 1:10 β-mercaptoethanol). The samples were heated at 95 °C for 5 min at 350 rpm. Proteins were separated by gel electrophoresis with 8%, 10% or 14% SDS-PAGE gels and transferred to polyvinylidene difluoride (PVDF) membranes. After transfer, membranes were blocked with 5% BSA in TBST (20 mM tris, 140 mM NaCl, 0.1% (*v*/*v*) Tween®-20, pH 7.4) for 1 h at RT and then incubated overnight with the respective antibodies in 5% BSA TBST at 4 °C. Membranes were washed three times with 1× TBST and incubated for 1 to 2 h at RT with the secondary-HRP-conjugated antibodies in 5% BSA-TBST. After three times of washing with TBST, membranes were developed by ECL detection.

### 2.8. Western blot Analysis Using Zn^2+^-Phostag^TM^-Gel Electrophoresis

Western blot samples were prepared with 3× Laemmli buffer for phostag (200 mM tris, 15% (*v*/*v*) glycerol, 6% (*w*/*v*) SDS, 2% (*w*/*v*) bromphenol blue, 1:10 β-mercaptoethanol) in the absence of EDTA. The samples were heated at 95 °C for 5 min. Proteins were separated according to their phosphorylation ratio using 6% (*v*/*v*) acrylamide phostag gels. The gels did not contain SDS, but contained phostag^TM^ compound (35 µM, depending on the protein of interest) and ZnCl_2_ (69 µM) in the separating gel. Phostag running buffer was used for gel electrophoresis (0.1 M tris, 0.1 M MOPS, 0.1% (*w*/*v*) SDS, 5 mM sodium bisulfite, pH 7.8). Prior to protein membrane transfer, gels were washed twice for 10 min with transfer buffer containing 1 mM EDTA, to remove the Zn^2+^-ions. A third washing step was performed with 1× transfer buffer without EDTA before the proteins were transferred to polyvinylidene difluoride (PVDF) membranes using a transfer buffer for phostag (25 mM tris, 192 mM glycine, 10% (*v*/*v*) methanol, 5% (*w*/*v*) SDS, pH 8.4).

### 2.9. Phosphorylation of GST-ARPP19 or HisENSA with Recombinant Kinases for Western Blot Analysis

Recombinant GST-ARPP19 (76 nM) or HisENSA (WT or S109 mutants, 76 nM) was incubated with active recombinant MASTL kinase (4 µM) or with PKA C-subunit (27 µM) or PKGIβ (16 µM plus 5 µM of 8-bromo-cGMP sodium salt), 1 mM ATP or thio-ATP in kinase dilution buffer (5 mM MOPS, 5 mM MgCl_2_, 1 mM EGTA, 0.4 mM EDTA, 0.05 mM DTT, 100 ng/µL BSA, pH 7.2). For the consecutively phosphorylation experiments, the second kinase was added 20 min after the first kinase and western blot samples were taken before (0 min) and after 5, 10, 20, 25, 30 and 40 min of incubation at 30 °C. For western blot analysis of single phosphorylation sites, samples were taken before (0 min) and after 0.5, 2 and 10 min (for HisENSA) or 5, 10 and 20 min (GST-ARPP19) of incubation at 30 °C.

### 2.10. Phosphorylation of GST-ARPP19 or HisENSA with Recombinant Kinases for Dephosphorylation Experiments in Platelet Lysates and for PP2A Phosphatase Activity Assay

Recombinant GST-ARPP19 or HisENSA were phosphorylated as described in 2.9. For western blot analysis, samples were taken before (0 min) and after 20 min of incubation. The phosphorylation reaction was stopped with 6 mM EDTA. For the PP2A activity assay, samples were desalted and buffer was exchanged with Zeba^TM^spin desalting columns (7K MWCO, 0.5 mL) to 5× PP2A reaction buffer (250 mM imidazole, 1 mM EGTA, 0.1% (*v*/*v*) 2-mercaptoethanol, 0.5 mg/mL BSA, pH 7.2).

### 2.11. Phosphorylation of Recombinant HisENSA and GST-ARPP19 in Platelet Lysates

Kinase phosphorylation buffer (10×: 100 mM HEPES, 50 mM MgCl_2_, 10 mM DTT, 0.2 mM EDTA, pH 7.2) and 1 mM ATP was added 1:10 to platelet lysates. As controls, lysates were incubated in the absence or presence of OA (2 µM or 2 nM). The reaction was started with addition of recombinant HisENSA (250 nM) or GST-ARPP19 (87 nM). Western blot samples were taken directly after protein addition (0 min), after 3, 10 and 30 min or after 10, 20 and 40 min of protein incubation at 30 °C, in 3× Laemmli buffer and heated for 5 min at 95 °C.

### 2.12. Dephosphorylation of GST-ARPP19 in Platelet Lysates

Isolated human platelets were pelleted and lysed in lysis buffer (50 mM tris, 150 mM NaCl, 1 mM EDTA, 0.1 mM EGTA, 0.25% (*v*/*v*) NP-40, 30 ng/µL BSA, pH 7.4) with 50% (*v*/*v*) volume of HEPES buffer to a final concentration of 1.5 × 10^9^ platelets/mL. GST-tagged ARPP19 (62.3 nM final concentration) phosphorylated by PKA at S104 or MASTL at S62 was added. Western blot samples were taken directly after protein addition (0 min) and after 5, 15 and 30 min of incubation at 37 °C, respectively. OA (2 nM), thio-phosphorylated GST-ARPP19 (62.3 nM final concentration; thiopS62 GST-ARPP19 for pS104 GST-ARPP19 dephosphorylation; thiopS104 GST-ARPP19 for pS62 GST-ARPP19 dephosphorylation) or non-phosphorylated GST-ARPP19 (62.3 nM) served as controls. All experiments were performed in the presence of 5× PP2A reaction buffer (250 mM imidazole, 1 mM EGTA, 0.1% (*v*/*v*) 2-mercaptotethanol, 0.5 mg/mL BSA, pH 7.2). 

### 2.13. Ser/Thr-Protein Phosphatase Inhibition by OA in Intact Human Platelets

Washed human platelets were incubated in the presence of 1 mM Ca^2+^ with OA (50 nM, 200 nM, 2 µM or 10 µM) or vehicle control (0.1% (*v*/*v*) or 0.25% (*v*/*v*) EtOH) at 37 °C for up to 40 min. Western blot samples were taken directly after OA/vehicle addition (0 min) and after 10, 20, 30 and 40 min of OA/vehicle addition into 3× Laemmli buffer and heated for 5 min at 95 °C.

### 2.14. PKA/PKG Effects in Intact Human Platelets

Washed human platelets (2 × 10^9^ platelets/mL) were incubated with 5 nM iloprost (PKA) or 10 µM riociguat (PKG) or kept resting (--) at 37 °C. Western blot samples were taken directly after (0 s) and 15, 30, 120 and 300 s after iloprost addition or 120, 300 and 600 s after riociguat addition into 3× Laemmli buffer and heated for 5 min at 95 °C.

### 2.15. Colorimetric Ser/Thr/PP2A Phosphatase Activity Assay

To assess PP2A activity in platelet lysates, a colorimetric molybdate-based Ser/Thr phosphatase assay was used according to the manufacturer’s protocol (Promega Serine/Threonine Phosphatase Assay System V2460). Platelet lysate was generated as described in 2.6. and free phosphate was removed using the spin columns provided by the assay system. Platelet count was adjusted to 1 × 10^8^ platelets/mL with lysis buffer (50 mM tris, 150 mM NaCl, 1 mM EDTA, 0.1 mM EGTA, 0.25% (*v*/*v*) NP-40, pH 7.4; cOmplete^TM^ protease inhibitor cocktail). 35 µL platelet lysate were added per well to start the reaction and incubated with phosphopeptide in PP2A reaction buffer for 60 min at 37 °C. 0 min control samples were stopped immediately after lysate addition with a mixture of molybdate dye/additive (50 µL/well). After 60 min samples were also stopped, the reaction was incubated additional 15 min at RT in the dark. For inhibitory experiments, OA, fostriecin, tautomycetin or recombinant proteins were added to the platelet lysate. As recombinant proteins were in 5× PP2A reaction buffer (250 mM imidazole, 1 mM EGTA, 0.1% (*v*/*v*) 2-mercaptoethanol, 0.5 mg/mL BSA, pH 7.2), the amount of additional 5× PP2A reaction buffer was adjusted, according to the amount of added recombinant protein. P2A activity was measured by absorbance of 630 nm using a 96-well plate reader (Dynex Opsys MR^TM^, Tarporley, CW6 9BL UK).

### 2.16. Light Transmission Aggregometry

Washed human platelets were adjusted to 2 × 10^8^/mL with HEPES-buffer including 1 mM CaCl_2_ and pre-incubated with OA (50 nM; 200 nM or 1 µM) or vehicle control (0.01% or 0.25% (*v*/*v*) EtOH) for 10 min at 37 °C. Alternatively, washed human platelets were pre-incubated with tautomycetin (20 µM or 30 µM) or vehicle control (1.5% (*v*/*v*) DMSO) for 10 or 30 min at 37 °C. Aggregation was started by addition of α-thrombin (0.05 U/mL or 0.1 U/mL final concentration) and monitored for 5 min at 37 °C under stirring (1000 s^−1^) using a photometric aggregometer (Apact 4S Plus; DiaSys, Flacht, Germany). Platelet aggregation was expressed as % of aggregation, which represents % of light transmission.

### 2.17. (Phospho-)Proteomic Sample Measurement

Washed human platelets were incubated with buffer (control), sodium S-nitrosocysteine (SNC, 5 µM for 2 min), sodium nitroprusside (SNP, 5 µM for 2 min), DEA-NO (5 µM for 2 min) or riociguat (10 µM for 5 min) at 37 °C. After incubation, samples were stopped by the addition of 4× SDS/lysis buffer (50 mM tris, 150 mM NaCl, 4% (*w*/*v*) SDS, pH 7.5), shock frozen in liquid nitrogen and analysed by quantitative phosphoproteomics as described [40]. The fold stimulation of phosphorylation of various phosphosites compared to control is shown as average ratio.

### 2.18. Statistical Analysis

Experiments were performed at least three times with at least three different healthy donors when platelet samples were involved. Data are presented as means ± standard deviation (SD). Statistical analysis was performed using GraphPad Prism 8 for Windows (GraphPad Software, San Diego, CA, USA). One-way ANOVA multiple comparisons test was used for comparison of more than two groups. *p* < 0.05 was considered as significant.

## 3. Results and Discussion

### 3.1. The PP2A Inhibitors ENSA and ARPP19 are Present in Human Platelets and Phosphorylated by Both PKA and PKG

In our earlier proteomic studies, we detected significant expression of both ENSA and ARPP19 in the small anucleate human platelets [34]. Subsequently, in our analysis of the iloprost/cAMP-stimulated phosphoproteome of human platelets, more than 130 cAMP/PKA regulated phosphoproteins were detected including ENSA (phosphorylated at S109) and ARPP19 (at S104) [35]. Platelets are strongly inhibited by both cAMP- and cGMP-elevating agents mediated by the corresponding cAMP- and cGMP-dependent protein kinases (PKA/PKG), which have overlapping specificity [18,19]. Therefore, we investigated the effect of the cGMP/PKG–pathway on ENSA/ARPP19 phosphorylation using various NO-donors [*S*-nitrosocysteine (SNC), sodium-nitroprusside (SNP), diethylamine NONOate (DEA-NO)] and the soluble guanylyl cyclase (sGC)-stimulator riociguat in comparison to the iloprost (cAMP) pathway, using vasodilator-stimulated phosphoprotein (VASP) as established PKA/PKG substrate and marker. Both ENSA (at S109) and ARPP19 (at S104) were strongly phosphorylated (several-fold stimulation over basal level, similar to VASP S239) in response to different cGMP-elevating agents as well as in response to the cAMP-elevating iloprost (Table 1). We also noted that both cAMP- and cGMP-elevating conditions reduced the basal phosphorylation of a second ENSA phospho-site, S67, (Table 1).

Previously, we demonstrated that under these conditions riociguat had no detectable effect on cAMP/PKA in human platelets [41]. Therefore, we performed a second, comprehensive phosphoproteomic analysis of iloprost- and riociguat–treated human platelets with three biological replicates. These phosphoproteomic data demonstrated that both ENSA (at S109) and ARPP19 (at S104) are phosphorylated in response to cAMP/PKA as well as to cGMP/PKG stimulation in intact human platelets, similar to VASP S239 (Table 2).

Phosphorylation of the PKA-preferred site of VASP, S157, was also detected but required a special protease treatment (subtilisin) for detection by phosphoproteomics. It is of special interest for this study that phosphorylation of the serine/threonine protein phosphatase 2A (PP2A) at its regulatory B56δ subunit (S573) was also detected for the iloprost/cAMP pathway and as well but less phosphorylated for the riociguat/cGMP pathway. Others previously showed that the PKA phosphorylation of PP2A B56δ at S573 activates this special PP2A heterotrimer, leading to a decrease in protein phosphorylation [42]. We also confirmed this important B56δ S573 phosphorylation in our experiments with iloprost-/riociguat-treated human platelets, using the specific phospho-antibody as described [42], see Figure S1.

**Figure 2:**
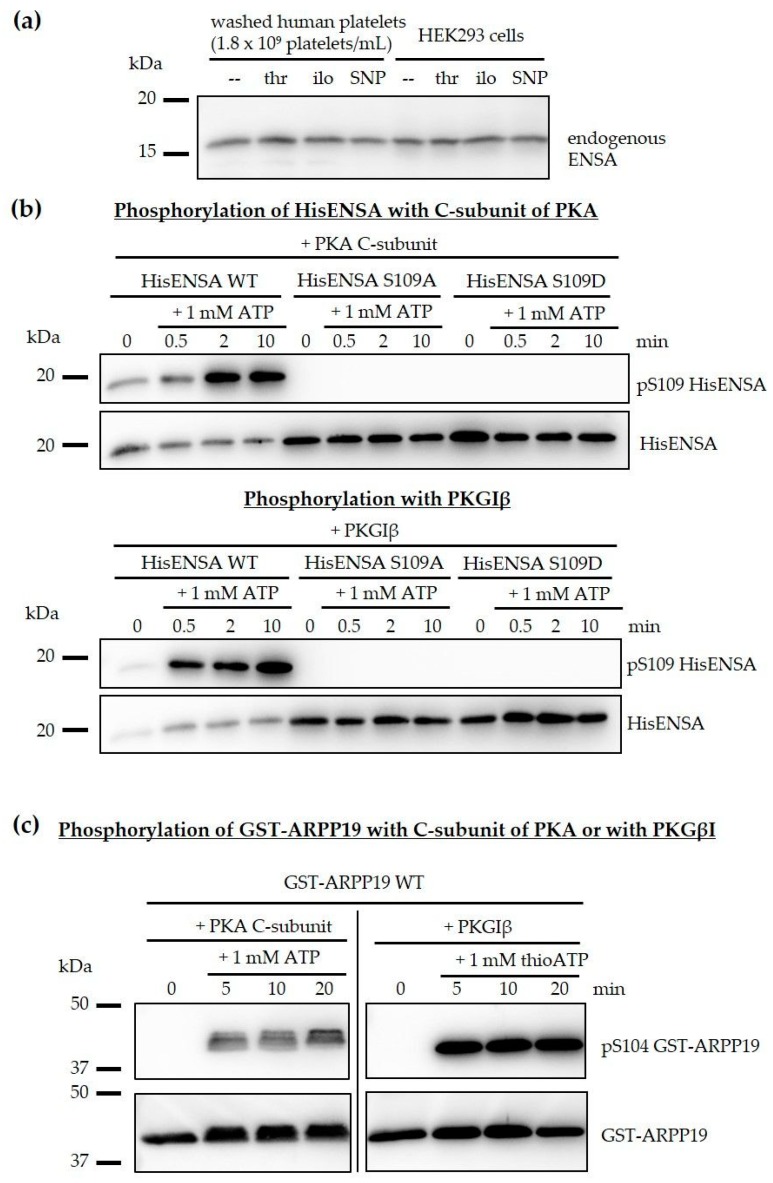
Human ENSA and ARPP19 are substrates of both PKA and PKG. (**a**) The molecular weight of endogenous ENSA in human platelets and HEK293 cells is similar and is not affected by different treatments (thrombin (thr), iloprost (ilo) or sodium nitroprusside (SNP)). (**b**) Recombinant HisENSA wildtype and phosphosite mutants S109A and S109D were phosphorylated in the presence of the C-subunit of PKA or of PKGIβ (with 5 µM cGMP for PKG) and 1 mM ATP. Samples were taken in Laemmli buffer before (0 min) and after 0.5, 2 and 10 min of ATP addition and analysed by immunoblotting. HisENSA S109A and S109D mutants were not phosphorylated confirming S109 is the phosphorylated amino acid. (**c**) Recombinant GST-ARPP19 was phosphorylated in the presence of PKA C-subunit or PKGIβ (and 5 µM cGMP) and 1 mM ATP. Samples were taken in Laemmli buffer before (0 min) and after 5, 10 and 20 min of ATP addition. The western blots showing HisENSA and GST-ARPP19 phosphorylation are representative for n = 3 independent experiments.

Our phosphoproteomic data show that both ENSA and ARPP19 are phosphorylation targets in response to the platelet inhibitory pathways cAMP and cGMP. It is indeed surprising to find the important phosphoproteins and cell cycle regulators ENSA and ARPP19 in non-dividing human platelets. We therefore confirmed our proteomic data via western blot analysis of washed human platelets and HEK293 cells using a well-established anti-ENSA antibody. Both human platelets and human HEK293 cells contained one ENSA species, (apparent 17 kDa in SDS-PAGE), independent of the experimental conditions (Figure 2a). We also cloned, expressed and purified human ENSA of the known canonical sequence to study the direct PKA-/PKG-specific phosphorylation of human recombinant HisENSA (including S109A and S109D phosphosite mutants) and, in comparison, recombinant GST-ARPP19 using pure PKA (C-subunit) and PKG (Figure 2b,c).

HisENSA and GST-ARPP19 were strongly phosphorylated by both PKA and PKG, which was abolished by ENSA S109A or S109D mutations. Other data showed that the PKA- and PKG-induced phosphorylation of ARPP19 S104 resulted in a complete shift of GST-ARPP19 in phostag-gels, indicating stoichiometric phosphorylation of ARPP19 at S104.

All data obtained with intact human platelets and with recombinant proteins proved that ENSA and ARPP19 are not only PKA but also excellent PKG targets. In fact, the extent of S109 ENSA phosphorylation in response to various cGMP-elevating platelet inhibitors (Table 1 and Table 2) compared to VASP 239 places especially ENSA among the best PKG targets studied. In contrast, the iloprost/cAMP pathway was more effective than the riociguat/cGMP pathway with respect to the phosphorylation of PP2A-B56δ (PPP2R5D) at S573, an established PKA substrate [42]. It is of special interest that both cGMP- and cAMP-elevating platelet inhibitors increased the phosphorylation of S109 ENSA while they decreased S67 ENSA phosphorylation (Table 1). Therefore, the properties of S67 ENSA and S62 ARPP19 phosphorylation in platelets were addressed next. 

### 3.2. Phosphorylation of ENSA S67/ARPP19 S62 by a MASTL-Related Protein Kinase in Human Platelets

**Figure 3:**
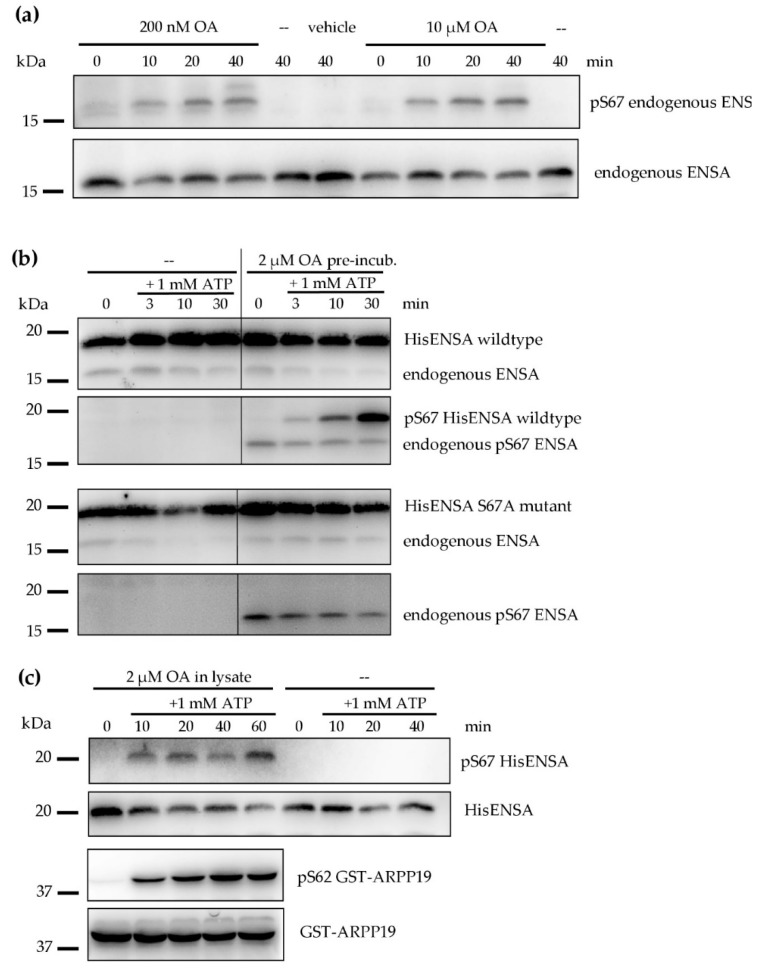
Effect of okadaic acid (OA) treatment on the phosphorylation of endogenous ENSA (S67) in intact human platelets and on the phosphorylation of added HisENSA (S67)/GST-ARPP19 (S62) in platelet lysates. (**a**) Isolated human platelets (2 × 10^9^ platelets/mL) were incubated with 200 nM or 10 μM of OA or vehicle (0.1% (*v*/*v*) ethanol) for 0, 10, 20 and 40 min. Both OA concentrations induced a S67 phosphorylation of endogenous ENSA, with noticeable effects already after 10 min. (**b**) S67 HisENSA phosphorylation was only detectable when OA was added to intact platelets or platelet lysates. Intact platelets were pre-incubated without or with 2 µM OA and lysed afterwards. HisENSA and 1 mM ATP were added to the platelet lysate. Samples were taken in Laemmli buffer before (0 min) and after 3, 10 and 30 min of 1 mM ATP addition. In contrast to endogenous ENSA and added HisENSA(wt), the added S67A HisENSA mutant was not phosphorylated, serving as important negative control. (**c**) HisENSA or GST-ARPP19 as well as 1 mM ATP were added to platelet lysate without (only with HisENSA) or with 2 µM OA in the lysate. Samples were taken before (0 min) and after 10, 20, 40 and 60 min of 1mM ATP addition. The western blots are representative for *n* = 3 independent experiments.

As introduced earlier, ENSA and ARPP19, when phosphorylated at S67/S62, strongly inhibit certain holoenzymes of PP2A. Considering this important functional role of S67 ENSA/S62 ARPP19 phosphorylation in other cell systems such as *Xenopus* oocytes and our preliminary detection of these phosphosites in human platelets by phosphoproteomics (Table 1), we investigated the possible S67 ENSA/S62 ARPP19 phosphorylation in human platelets and their lysates. With the Cell Signaling^®^ anti-pS67 ENSA/anti-pS62 ARPP19 phospho-antibody, which recognizes the conserved pS67 ENSA site as well as pS62 ARPP19 (sequence 100% similar), positive signals were initially detectable only in HEK293 cell samples (Figure S2, used as positive control). However, it has been reported for other systems (*Xenopus* oocytes, HeLa cells) that Gwl/MASTL-phosphorylated ENSA/ARPP19 are rapidly dephosphorylated by PP2A, which could be completely prevented by the PP2A inhibitor okadaic acid (OA) [43,44]. In our experiments pre-incubation of intact platelets with OA at low (200 nM) and high (10 µM) concentrations induced a similar time–dependent phosphorylation of endogenous ENSA S67 (Figure 3a). This ENSA pS67 band may include some ARPP19 pS62 since the phospho-antibody recognizes both ENSA pS67 and ARPP19 pS62, and platelets contain about 3-fold more ENSA than ARPP19 (Table 2). 

We then performed phosphorylation experiments with lysates from human platelets, pre-incubated with 2 µM of OA or vehicle control. Only lysates from OA pre-treated platelets (but not controls) supported a time–dependent S67-phosphorylation of added recombinant HisENSA wildtype (Figure 3b, upper part) whereas a S67A HisENSA mutant was negative (Figure 3b, lower part). This experiment additionally validated the phosphorylated site and the phospho-antibody studied. A consistent phosphorylation of endogenous ENSA at S67 already in the beginning of the lysate incubation can also be noted, probably due to the intracellular OA pre-incubation effects before lysis. OA added to lysates of untreated platelets also induced the phosphorylation of S67 HisENSA, and as well of S62 GST-ARPP19 (Figure 3c).

A control without OA showed no S67 HisENSA signal after 40 min of incubation. These results clearly indicate the presence of a protein kinase in human platelets that is able to phosphorylate ENSA and ARPP19 at S67 or S62, respectively. The protein kinase seems to be active in intact platelets as well as in platelet lysates but its activity can only be detected, when an ENSA/ARPP19-specific serine/threonine protein phosphatase (most likely PP2A) is inhibited.

These experiments with intact human platelets and their lysates establish for the first time a significant activity of a protein kinase, which phosphorylates endogenous and/or added HisENSA/GST-ARPP19 at the sites S67/S62 known in other systems to be responsible for PP2A inhibition. The absent S67 ENSA phosphorylation under basal conditions but clear detection when PP2A is inhibited (Figure 3) suggests that the protein kinase responsible for S67 ENSA phosphorylation in the platelets is inhibited by PP2A-mediated dephosphorylation. In addition/or alternatively a very fast dephosphorylation of pS67 ENSA under basal and other conditions in human platelets is mediated by PP2A and inhibited by OA.

In mammalian cells, the only two protein kinases with the capacity to phosphorylate S67 ENSA/S62 ARPP19 (at the identical phosphosite motif KGQKYFDSGDYNMAK) described so far are MASTL (microtubule-associated serine/threonine kinase-like, the human orthologue of the Greatwall kinase, Gwl) [45,46] and the related MAST3 kinase [47,48]. However, in our proteomic studies of human platelets [34] and related studies of murine platelets [49] neither MASTL nor MAST3 were detected. Also, a comprehensive analysis of the human and platelet transcriptome did not detect significant expression levels of MASTL or MAST3, in contrast to significant expression levels of ENSA and ARPP19 [50]. At present, there are two possible explanations for our data on MASTL kinase protein (negative) and MASTL activity (positive) in human platelets. Despite the negative protein expression data so far, there are perhaps low, not yet detectable MASTL/MAST3 levels in platelets, which mediate the observed S67 ENSA/S62 ARPP19 phosphorylation. Alternatively, platelets may contain other MASTL-related protein kinases, which catalyse ENSA/ARPP19 phosphorylation, since human platelets express, at the protein level, about 150 different protein kinases from all classes [34,51]. Clearly, it will be important to elucidate the identity of the S67 ENSA/S62 ARPP19 protein kinase and its regulation within platelets (see also our limitation paragraph, end of the discussion). 

It is of considerable interest that the discovery of MASTL as human Gwl orthologue [45] also defined a MASTL E167D mutation in humans, which is associated with thrombocytopenia [45,46,52]. Very recently, a megakaryocyte specific MASTL mutation (E167D knock-in) and a complete MASTL KO in mice was reported with a reduced platelet count and decreased half-life of platelets in MASTL KO and mutant knock-in mice. In addition, increased annexinV-levels, probably associated with platelet apoptosis or platelet activation, increased bleeding times and defective clot retraction were observed [53]. Overall, this MASTL mutation (E167D) was considered a gain of MASTL kinase function, leading to a stronger inhibition of PP2A. However, the study did not address the presence of MASTL protein or activity in platelets.

### 3.3. Phosphorylation of HisENSA and GST-ARPP19 by Human MASTL (S67/S62) and by PKA C-Subunit/ PKGIβ (S109/S104) or Combinations

**Figure 4:**
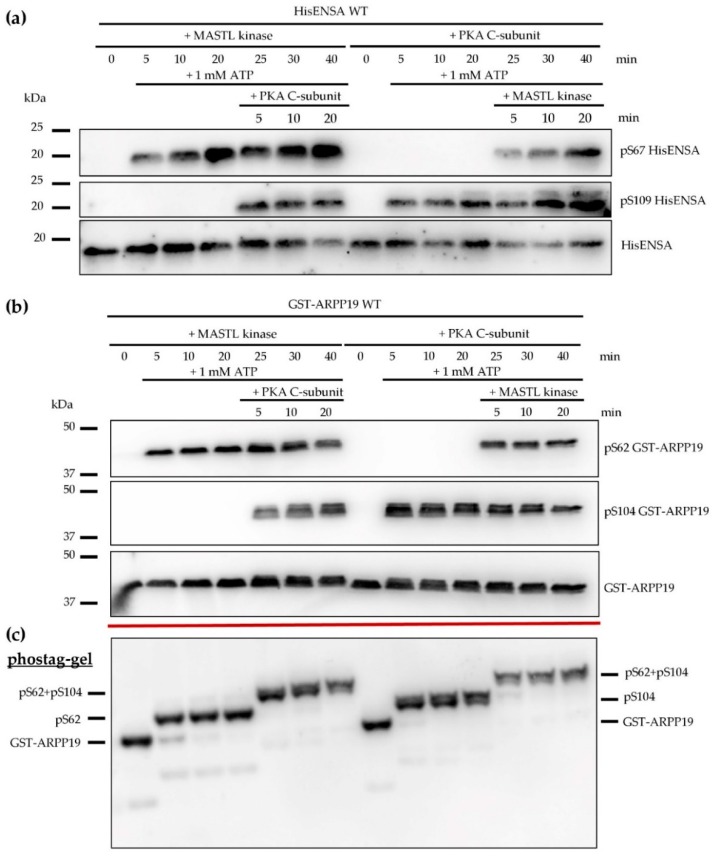
Site-specific phosphorylation of HisENSA or GST-ARPP19 by MASTL and PKA in combination. (**a**) HisENSA was phosphorylated consecutively in the presence of MASTL, C-subunit of PKA and 1 mM ATP. One of the kinases was added first, the second kinase after 20 min. Samples were taken after 5, 10, 20, 25 (5 min after addition of second kinase), 30 and 40 min of incubation and stopped by Laemmli buffer. The western blots are representative for n = 3 independent experiments. (**b**) GST-ARPP was phosphorylated consecutively in the presence of MASTL and C-subunit of PKA and 1 mM ATP. One of the kinases was added first, the second kinase after 20 min. The reaction was stopped after 5, 10, 20, 25 (5 min after addition of second kinase), 30 and 40 min by Laemmli buffer. (**c**) Corresponding phostag-gel of the same samples as shown in (**b**). The 6% acrylamide-phostag-gel (with 35 µM phostag) separated the proteins by their number of phosphorylated sites. The western blots are representative for *n* = 3 independent experiments.

An important goal of this project was to investigate the possible inhibitory effect of ENSA and ARPP19 on serine/threonine protein phosphatases in human platelets. As prerequisite, the phosphorylation of the recombinant HisENSA and GST-ARPP19 proteins was studied in more detail (Figure 4).

As already shown, PKA and PKG strongly phosphorylated HisENSA S109 and GST-ARPP19 S104. The ENSA S109 phosphorylation was abolished with ENSA S109A/D mutants but not with the S67A ENSA mutant (Figure S3). Importantly, PKA and PKG phosphorylation of GST-ARPP19 caused a complete time-dependent mobility shift of GST-ARPP19 in phostag-gels (shown for PKA C-subunit in Figure 4c; data for PKG similar shown in Figure S4b) demonstrating stoichiometric (complete) phosphorylation of the PKA/PKG phosphosite under these conditions, as phostag-gels are separating the proteins by their number of phosphorylated sites and not by their molecular weight.

This phostag-gel analysis worked very well with GST ARPP19 but did not work with HisENSA, maybe due to interference of the His-Tag with Zn^2+^-ions of the phostag-gel. Recombinant human MASTL kinase phosphorylated HisENSA and GST-ARPP19 strongly and time-dependently at a site recognized by the pS67 ENSA/pS62 ARPP19 antibody. With GST-ARPP19, MASTL phosphorylation caused a time-dependent complete mobility shift of GST-ARPP19 in phostag-gels, demonstrating stoichiometric phosphorylation of the MASTL-site S62. Next, consecutive phosphorylation of HisENSA or GST-ARPP19 by kinase combinations (MASTL+PKA, Figure 4b,c; MASTL+PKG, Figure S4a) were investigated. The two important phosphorylation sites (MASTL: S67/S62; PKA/PKG: S109/S104) were effectively phosphorylated by the responsible kinases without interference by the other kinase. Specifically, MASTL had no major effect on PKA/PKG-mediated ENSA/ARPP19 phosphorylation, and PKA/PKG had no major effect on the MASTL-mediated ENSA/ARPP19 phosphorylation. This was clearly demonstrated by phostag-analysis of ARPP19 phosphorylation and its two independent shifts by S62 and S104 phosphorylation (Figure 4b,c, Figure S4b). At present, minor interactions between both kinases cannot be excluded since detailed kinetics analyses were not performed. Also, we cannot rule out interferences of the protein tags used in our experiments with ENSA/ARPP19 proteins. However, we have no evidence for this, and such tagged ENSA/ARPP19 proteins have been successfully used by many investigations in this field. Finally, the effects may be different in intact cells and may be cell type-/system-specific, as discussed below. 

Mochida reported in 2014 the different phosphorylation sites of ENSA in *Xenopus* oocytes and their influence on PP2A inhibition [31]. Whereas human ENSA (and ARPP19) apparently only have two major phosphorylation sites (S67/S62 for MASTL, S109/S104 for PKA), *Xenopus* ENSA and ARPP19 have a third site, T28 for ENSA and S28 for ARPP19. It was concluded that the phosphorylation of all three sites influences PP2A inhibition. However, pS67 ENSA was shown to be the most potent PP2A inhibitor interacting with both B55- and C-subunits and blocking the catalytic centre of PP2A. Mochida calculated the IC_50_ values for pENSA and PP2A B55 inhibition for his experiments using *Xenopus* oocytes and obtained an IC_50_ of 0.47 nM for pS67 ENSA, an IC_50_ of 0.52 nM for pS67+pS109 ENSA and much higher values for pS109 ENSA alone [31]. PKA phosphorylation of *Xenopus* ENSA S109 had little effect in these systems.

Also in *Xenopus* oocytes, PKA did not affect Gwl-induced ARPP19 phosphorylation nor the ability of Gwl-phosphorylated ARPP19 to inhibit PP2A B55δ. The authors concluded that the effect of S67 phosphorylation was dominant over the negative-function of S109-phosphorylation [54], in agreement with earlier studies by others [31].

Andrade et al. showed in 2017 that brain ARPP16 phosphorylation sites (the shorter isoform of ARPP19) were reciprocally phosphorylated when forskolin (activator of adenylate cyclase and thus of PKA) was added to striatal slices [47,48]. Forskolin induced a reduced phosphorylation of the MASTL/MAST site S46 (similar to S62 ARPP19/S67 ENSA) and stimulated S88 phosphorylation via PKA (similar to PKA sites S104 ARPP19/S109 ENSA). In that paper, several mechanisms were discussed to explain how S46 ARPP16 phosphorylation could be blocked resulting in reduced PP2A inhibition:

- PKA phosphorylation of ARPP16 could interfere with the extent of PP2A inhibition by pS46 ARPP16
- PKA could phosphorylate and inhibit the ARPP16 S46 kinase MAST3
- Forskolin-stimulated PKA could activate PP2A by phosphorylation of PP2A B56δ [42] resulting in reduced S46 ARPP16 phosphorylation

Some of these effects may perhaps be specific for brain, as was recently reviewed [55]. However, in our phosphoproteomic analysis, we clearly detected PP2A B56δ S573 phosphorylation in human platelets (Table 2) in response to iloprost (cAMP system) and riociguat (cGMP system), which we were able to confirm using phospho-specific antibodies (Figure S4).

In our studies, PKA-phosphorylation of ENSA/ARPP19 did not affect their properties as MASTL substrates or in other in-vitro functions tested so far, but further work is required here. In intact platelets (Table 1) and transfected HEK293 cells (Figure S2) ENSA S109 was strongly phosphorylated (transfected ENSA protein stronger than endogenous) when PKA was activated and, at the same time, down-regulation of S67 ENSA phosphorylation was observed (stronger signal with transfected ENSA, weaker with endogenous ENSA) (Figure S2). Although our data with human platelets and the published results with murine striatum [47,48] are very similar (PKA reduced ENSA S67 or ARPP16 S62 phosphorylation, respectively), it will be necessary to identify the ENSA S67/ARPP19 S62 protein kinase in platelets for further mechanistic studies (see also the paragraph limitation). 

### 3.4. Serine/Threonine Protein Phosphatases in Human Platelets

The availability of recombinant ENSA and ARPP19 in various phospho-variants allowed the analysis of their effects on platelet serine/threonine protein phosphatases. Although the general importance of serine/threonine protein phosphatase including PP2A as signalling opponent of serine/threonine protein kinases has been recognized [25,26,56], the diversity and distinct regulatory mechanisms of serine/threonine protein phosphatases were only recently discovered, also supported by the complete description of the human protein kinome [57] and protein phosphatome [58]. With this background and our quantitative human platelet proteome data [34] it was possible to compile an overview of most serine/threonine protein phosphatases present in human platelets which include three catalytic subunits of PP1 and two catalytic subunits (α,β) of PP2A (Table S1). Table S1 also shows the intracellular concentrations of the catalytic subunits of serine/threonine protein phosphatases present in human platelets. It is remarkable that the major serine/threonine protein phosphatases such as PPM1, PP1, PP2A are present in human platelets at µM concentrations. This certainly is important for their catalytic capacity since most potential substrates have a similar intracellular concentration.

Whereas serine/threonine phosphatases were considered for a long time as unregulated components which ‘simply’ remove phosphates from serine/threonine phosphoproteins, it is now clear that serine/threonine protein phosphatases, and in particular PP1 and PP2A, are multimeric enzymes which are tightly controlled by regulatory subunits and additional activators and inhibitors [26]. 

Considering the enormous heterogeneity of PP2A composition and function, it was important to establish quantitative information on the expression level of PP2A subunits and ENSA/ARPP19 in human platelets, which is based on our proteomic databases (Table S2). We also estimated the intraplatelet concentration of these proteins, which is important for ENSA/ARPP19-PP2A interactions (see below). It is of interest that the PP2A A subunit concentration (1.67 µM) is higher than that of the two C subunits (1.07 µM) and similar to the concentration of all B subunits (1.73 µM) (Table S2). This indicates that there is no free PP2A C subunit in platelets, which agrees with observations in other cells and systems [26].

Human platelets contain the two catalytic subunits (α,β) of PP2A, only one of the two scaffolding A (α), two B55 subunits (α,δ), five B56 subunits (α,β,γ,δ,ε) and the PP2A activator, which is known to activate PP2A [59]. Interestingly, the distribution of PP2A isoforms in human platelets is similar to murine platelets, although some quantitative differences exist. For the scaffolding subunit A, only the α-isoform was found in human platelets, similar to other human cells [60]. The proteomic data suggest that 14 different PP2A trimeric holoenzymes and two different dimeric forms may exist in human platelets, perhaps even more due to splice variants of the regulatory subunits. The content of serine/threonine protein phosphatases in platelets and their subunit composition (especially of PP2A) is important to evaluate the following phosphatase assays. 

### 3.5. Effects of PP2A Inhibitors and MASTL-Phosphorylated HisENSA/GST-ARPP19 on Human Platelet PP2A Activity

**Figure 5:**
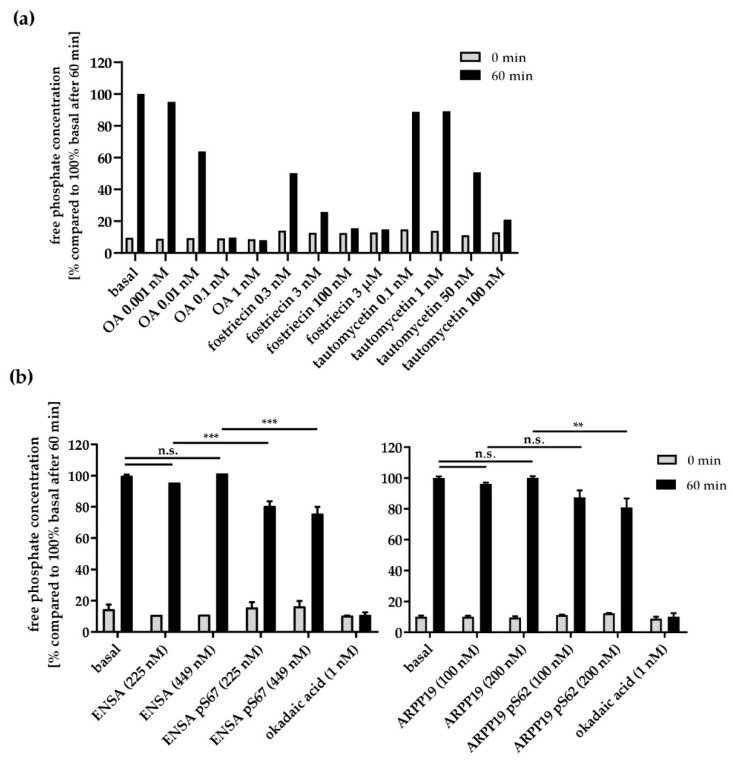
Effects of PP2A/PP1 inhibitors and of recombinant ENSA/ARPP19 proteins on Ser/Thr-protein phosphatase activities of platelet lysates analysed by a colorimetric peptide assay. (**a**) PP2A activity in lysates of human platelets after 0 or 60 min under basal conditions or in the presence of OA, fostriecin or tautomycetin (different concentrations). 0.1 nM and 1 nM of OA as well as 100 nM and 3 µM fostriecin completely inhibited PP2A activity in platelet lysates. 0.1 nM/1 nM tautomycetin had little effect in this assay, whereas 50 nM and 100 nM of the compound partially inhibited this PP2A activity. Data are expressed as percentage of free phosphate concentration compared to 100% basal free phosphate concentration after 60 min. Data are shown as mean of technical triplicates. (**b**) PP2A activity in lysates of human platelets after 0 and 60 min under basal conditions or in the presence of OA, recombinant HisENSA, HisENSA pS67, recombinant GST-ARPP19 and GST-ARPP19 pS62, respectively. Data are expressed as percentage of free phosphate concentration compared to 100% basal free phosphate concentration after 60 min, mean ± SD of three independent experiments. ** *p* < 0.01; *** *p* < 0.001; n.s.: not significant.

To evaluate the effects of S67/S62-phosphorylated ENSA/ARPP19 on PP2A activity of human platelets, we established a PP2A assay, which measures the release of free phosphate by the absorbance of a molybdate:phosphate complex [61]. This Ser/Thr-phosphatase assay was optimized for PP2A by selective assay conditions including a specific buffer, selective peptide substrate and appropriately desalted recombinant proteins and cell lysates (Figure 5).

This measured phosphatase activity was potently inhibited by OA (concentrations of 1 nM and even below) and by fostriecin (0.3–3.0 nM) (Figure 5a), in agreement with the reported IC_50_ of 0.1–0.3 nM OA or of 1–3 nM fostriecin for PP2A inhibition, respectively [56,62]. In contrast, the PP1 inhibitor tautomycetin (reported IC_50_ for PP1 ~0.5 nM, IC50_50_ for PP2A ~62 nM) [56,63] had inhibitory effects on the measured phosphatase activity only at concentrations of 50 nM and higher. These data established the conditions to study and compare the effects of known (low dose OA) and putative inhibitors of PP2A such as S67/S62-phosphorylated ENSA/ARPP19. Addition of both, HisENSA or GST-ARPP19 phosphorylated by MASTL at S67/S62 in vitro, resulted in a significant reduction of platelet PP2A activity (Figure 5b). In contrast, non-phosphorylated ENSA/ARPP19 proteins had no effect. As control, 1 nM OA resulted in complete inhibition of phosphatase activity. Although these results established for the first time a role for phosphorylated ENSA and ARPP19 in the inhibition of PP2A in human platelets, the effects observed were moderate (~20% inhibition). A likely explanation is that ENSA and ARPP19 target only certain B subunit containing PP2A heterotrimers present in human platelet lysates (see Tables S1 and S2). In contrast, OA binds directly to and inhibits all PP2A catalytic subunits, independent of the regulatory B subunits, and also inhibits structurally closely related serine/threonine protein phosphatases such as PP4/PP6 [62]. S67-/S62-phosphorylated ENSA/ARPP19 are thought to achieve their inhibitory effects via direct binding to a limited number of PP2A regulatory B-subunits (B55/B56 subfamily) [31,33,48,55]. However, there are very few studies available on the regulation of mammalian/human PP2A by the ENSA/ARPP family, and none for platelets and other blood cells. 

### 3.6. Ser-Phosphorylated ARPP19 is Both a Substrate for and Inhibitor of Platelet PP2A

*Xenopus* ENSA, when phosphorylated at S67 by Gwl kinase, is both a substrate (but poor one) and inhibitor of *Xenopus* PP2A [31,43]. This led to the concept that S67-phosphorylated ENSA is a PP2A ‘inhibitor by unfair competition’ and that PP2A could also inactivate its own inhibitor. Therefore, we tested whether S62- or S104-phosphorylated GST-ARPP19 are substrates and inhibitors of protein phosphatases in platelet lysates. We performed these experiments using GST-ARPP19 since we could prepare and monitor stoichiometric MASTL- or PKA/PKG–phosphorylated pS62 GST-ARPP19 or pS104 GST-ARPP19, respectively, (Figure 4, Figure S4), which could then be tested as PP2A substrates or inhibitors. 

**Figure 6:**
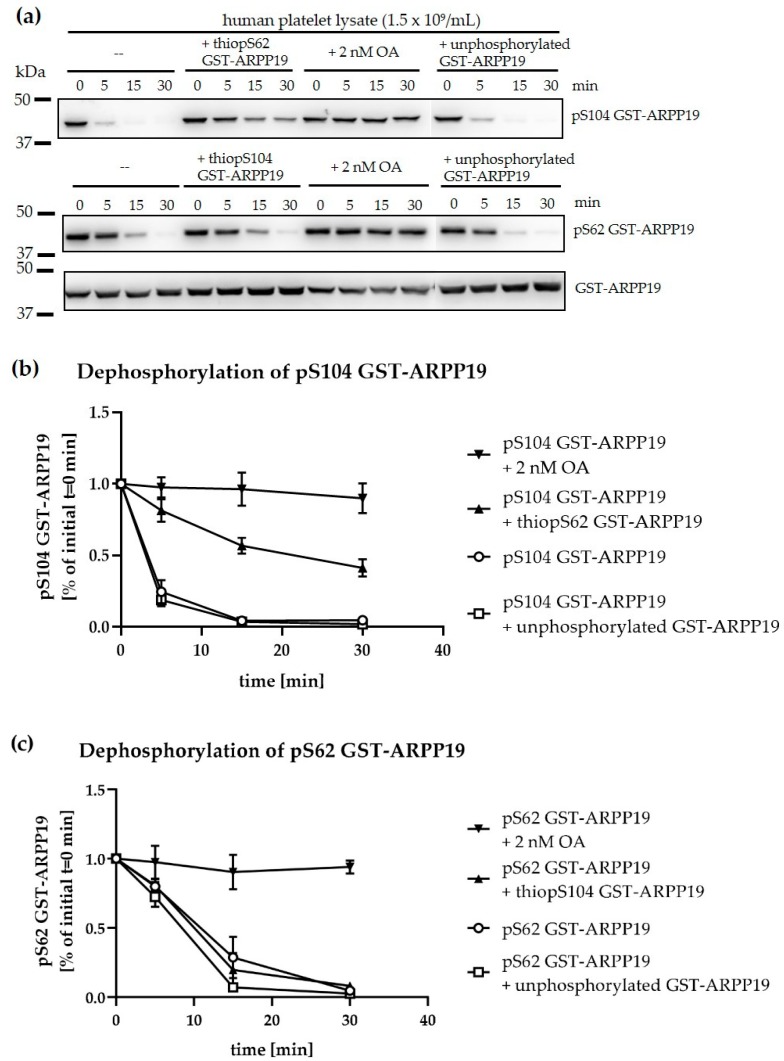
S62- or S104-phosphorylated GST-ARPP19 proteins are substrates of platelet PP2A, but only S62-phosphorylated GST-ARPP19 is a potent PP2A inhibitor. (**a**) GST-ARPP19 phosphorylated at S104 (up) or S62 (down) were incubated with human platelet lysates (obtained from 1.5 × 10^9^ platelets/mL) alone or in the presence of thio-pS62 GST-ARPP19, 2 nM OA, or non-phosphorylated GST-ARPP19 at 37 °C. Samples were taken in Laemmli buffer before (0 min) and after 5, 15 and 30 min of ARPP19 addition to lysate and analysed by immunoblotting using phosphospecific antibodies. Representative western blots are shown. (**b**) Dephosphorylation of PKA-phosphorylated (S104) GST-ARPP19 in human platelet lysates. The level of phosphorylation was densitometrically quantified and normalized to the 0 min control sample. Data are presented as means +/− SD of three independent experiments. (**c**) Dephosphorylation of MASTL-phosphorylated (S62) GST-ARPP19 in human platelet lysate. The level of phosphorylation was densitometrically quantified and normalized to the 0 min control sample. Data are presented as means +/− SD of three independent experiments.

Western blot analysis and quantification showed a fast and complete dephosphorylation of PKA-phosphorylated pS104 GST-ARPP19 (50% or more dephosphorylated at ~3 min, Figure 6a, upper panel, first four lanes and Figure 6b) and a slower, but also complete dephosphorylation (50% dephosphorylated at 10 min) of MASTL-phosphorylated pS62 GST-ARPP19 (Figure 6a, lower panels, first four lanes and Figure 6c) constitutively in lysates of washed human platelets. The addition of 2 nM OA to platelet lysates completely inhibited the dephosphorylation of both sites, indicating that this dephosphorylation is mediated by one of the PP2A protein phosphatases present in human platelets (Table S1).

Then we tested whether S62-phosphorylated GST-ARPP19 can serve as PP2A inhibitor. For this, we used thio-phosphorylated S62 GST-ARPP19, since this prevents dephosphorylation and inactivation of ENSA/ARPP proteins as reported for *Xenopus* oocytes, HeLa cells and brain cells [31,43,48]. In platelet lysates, thio-pS62 GST-ARPP19 potently diminished the dephosphorylation of pS104 GST-ARPP19, whereas unphosphorylated GST-ARPP19 had no effects (Figure 6a,b). On the other hand, thio-phosphorylated S104 GST-ARPP19 and unphosphorylated GST-ARPP19 did not influence the dephosphorylation of pS62 GST-ARPP19 (Figure 6a,c). These data indicate that MASTL- as well as PKA-phosphorylated pS62 GST-ARPP19/pS104 GST-ARPP19 are substrates of a platelet PP2A, sensitive to low doses of OA. Furthermore, pS62 GST-ARPP19 is a potent inhibitor as well as a substrate of these PP2A forms. The data fully agree with the concept of ‘inhibition by unfair competition’ [43] of platelet PP2A by S62/S67 phosphorylated ARPP19 and ENSA.

The original concept of ‘inhibition by unfair competition’ also established an important requirement for this mechanism to work in intact cells: S67-phosphorylated ENSA should be present in molar excess over PP2A-B55 in mitosis to account for the total absence of PP2A activity during M phase [43]. This actually was shown for *Xenopus* oocytes with an estimated intracellular concentration of 100–250 nM for PP2A-B55 and 500 nM–1 µM for ENSA, which could be phosphorylated at S67 up to 50%. It is of considerable interest that similar or even somewhat higher concentrations of these components are found in human platelets (1.73 µM all PP2A B subunits, 1.7 µM ENSA-ARPP19, Table S2). There is important evidence that the family of regulatory B subunits defines special properties of PP2A protein phosphatases, including ENSA/ARPP19 inhibitory effects, PP2A substrate specificity, interaction with additional proteins and subcellular localization [26,64,65,66,67]. Interestingly, mammalian cells have a much higher number of B subunit genes than lower eukaryotes [68]. Furthermore, phylogenetic research suggests that the expansion of the B subunit gene family was driven by the functional diversification of PP2A [69]. It is important to notice that human platelets contain the B55α, B55δ, B56α, B56β, B56γ, B56δ and B56ε PP2A subunits (Table S2), but only some of them (underlined) were shown so far to interact with *Xenopus* ENSA [31] or murine ARPP16 [48]. For human platelets, it will be very important now to establish which of the multiple platelet PP2A B subunits directly interacts with phosphorylated ENSA, ARPP19 or both. 

**Figure 7:**
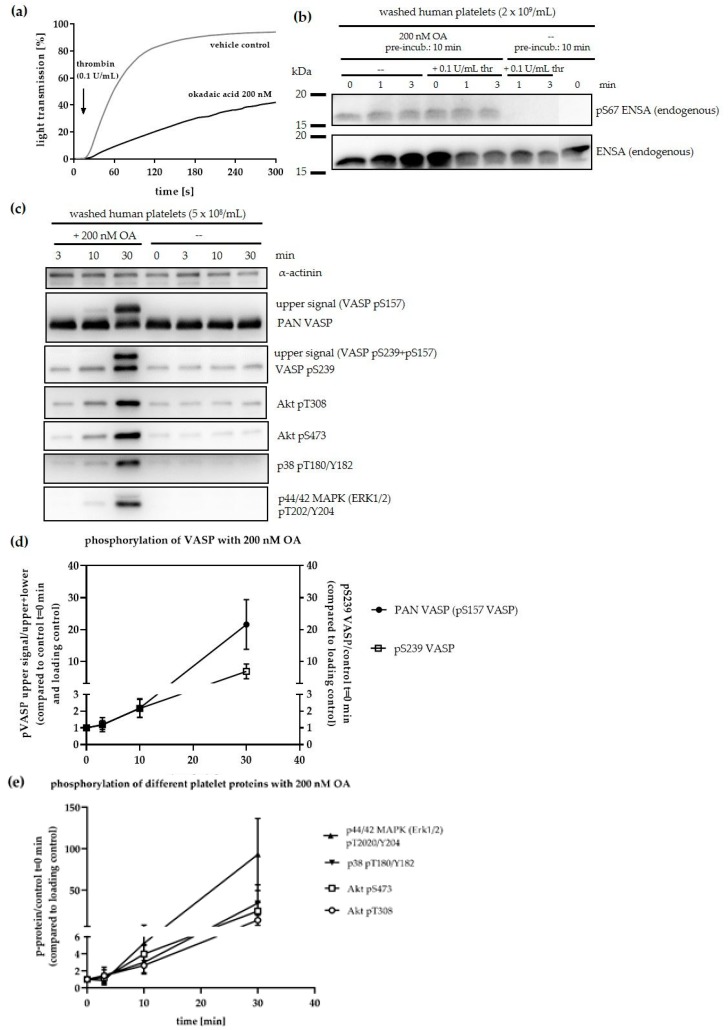
Inhibition of the PP2A family in human platelets affects platelet activation and multiple signalling pathways. (**a**) Washed human platelets (2 × 10^8^ platelets/mL) were incubated with 200 nM OA or vehicle control (0.01% (*v*/*v*) EtOH) for 10 min at 37 °C, platelets were activated with α-thrombin 0.1 U/mL (final concentration) and aggregation was measured for 5 min. Data are representative for 3 independent experiments. (**b**) Immunoblotting results of isolated human platelets pre-incubated without (--) or with 200 nM OA at 37 °C for 10 min, platelets were activated with α-thrombin 0.1 U/mL (final concentration) or kept resting and samples taken before (0 min) and 1 and 3 min after activation with Laemmli buffer. Data are representative for 3 independent experiments. (**c**) Representative immunoblotting results of isolated human platelets treated without (--) or with 200 nM OA at 37 °C. for 3, 10 and 30 min and stopped with Laemmli buffer. Phosphorylation of VASP, Akt, p38 and p44/42 MAPK were analysed by immunoblotting compared to the stable α-actinin signal as loading control. (**d**) Quantification of the phosphorylation of different proteins upon 200 nM OA incubation of intact human platelets. The level of phosphorylation was densitometrically quantified and normalized to the 0 min control sample. Data are presented as means +/− SD of three independent experiments. (**e**) Quantification of VASP phosphorylation upon 200 nM OA incubation of intact human platelets. The level of phosphorylation for pS239 VASP (detected by a pS239-phosphospecific antibody, sum of lower and upper band) was densitometrically quantified in comparison to the loading control and normalized to the 0 min control sample. pS157 VASP phosphorylation is visualized by the shift of VASP from the 46 kDa form (lower band) to the upper band (50 kDa) detected by a pan VASP antibody. Here, about 40% of VASP shifted to the upper band at 30 min OA treatment, indicating that 40% of VASP is S157 phosphorylated. This phosphorylation of VASP is shown here as ratio of the upper band compared to the total VASP signal in relation to each loading control. Data are presented as means +/-SD of three independent experiments.

It is therefore of importance to define both, inhibitors and substrates of PP2A in platelets. In contrast to protein kinases and their substrates/inhibitors, no reliable consensus sequence for serine/threonine protein phosphatases including PP2A have been established to date [26] in spite of considerable research efforts [65,70]. Nevertheless, multiple PP2A substrates have been studied and established within various processes such as intracellular signalling, cell cycle regulation, cell morphology and development as well as in brain function [23,55,71,72]. Because of the possible contribution of PP2A substrates to cell growth, cell survival and tumour suppression, phosphoproteins of the MAP kinase pathway and PI3K/Akt signalling have been particularly well studied [72,73,74]. For human platelets, very few studies have addressed PP2A substrates, even though, the PKA/PKG substrate and cytoskeletal protein VASP [75,76] and the p38 MAPK [36] were described as PP2A targets using OA and fostriecin as inhibitors of platelet PP2A. We therefore next studied the effect of OA on platelet activation/aggregation and on the state of phosphorylation of several known or putative PP2A substrates. We used 200 nM OA because it increased the phosphorylation of S67 ENSA already after 10 min incubation time reaching near-maximal effects at 20–40 min (Figure 3a). A 10 min incubation with 200 nM OA inhibited thrombin-induced aggregation of washed human platelets (Figure 7a; Figure S5a), which was associated with stable S67 phosphorylation of endogenous ENSA (Figure 7b). A 30 min incubation of intact human platelets with 200 nM OA strongly increased the phosphorylation of VASP (at S157 and S239), Akt (at T308 and S473), p38 (at T180/Y182) and p44/42 MAPK (Erk 1/2) (at T202/Y204) with initial effects observed within 10 min (Figure 7; Figure S6) after OA addition.

It needs to be considered that 200 nM OA used here with intact human platelets is relatively high compared to the PP2A inhibition by OA in cell free lysates (Ki 0.032 nM) and may affect PP1 (inhibition by OA in cell free lysates, Ki 150 nM) [56]. However, the conditions with intact cells (here intact platelets) are determined by additional factors. Although OA is membrane-permeant, it often requires concentrations of more than 50 nM to achieve significant effects within 30 min of incubation time [77]. A very important factor for OA effects in intact cells is also the concentration of the primary OA targets. In human platelets, we estimated here for the first time (see Tables S1 and S2) the concentration of PP2A catalytic subunits (α + β) as ~1 µM**,** that of PP1 catalytic subunits (α,β,γ) 7.1 µM. Considering these biochemical parameters, it appears reasonable that biochemical and functional OA-/PP2A-mediated effects with human platelets require incubation times of about 10-30 min and concentrations of 50–200 nM as used in our experiments with intact cells. Furthermore, it appears unlikely that these conditions affect PP1 since initially all OA entering platelets will be bound by the OA target with the highest OA affinity (PP2A) which acts like a sink (PP2A has a ~4.700 fold higher OA affinity compared to PP1). Only with higher concentrations or longer incubation times, other targets are hit when PP2A sites are saturated. We have also obtained supporting experimental data for this important topic. A 30 min incubation of only 50 nM OA with intact human platelets showed comparable effects for all phosphoproteins mentioned above (Figure S6). To rule out some participation of PP1 in these OA effects, we also compared the effects of both OA and tautomycetin (a strong PP1 inhibitor) on thrombin-induced shape change and aggregation of intact human platelets. Platelet shape change is mediated by myosin light chain phosphorylation, which is controlled by myosin light chain kinase (MLCK) and, as MLCK opponent, by myosin light chain phosphatase (MLCP), a specialized PP1 [13,78]. Inhibition of MLCP, for example by cGMP/PKG, strongly inhibits platelet shape change. In our experiments here, thrombin-induced activation and aggregation of human platelets was completely inhibited after 30 min of incubation with 30 µM tautomycetin, and shape change was inhibited as well (Figure S5b). The absence of a shape change clearly indicates inhibition of the MLCP (PP1) in human platelets [13]. In comparison, high amounts (1 µM) of OA completely inhibited platelet aggregation, but did not inhibit the PP1-mediated shape change (Figure S5a). Based on these results and on our other information on platelet PP2A and PP1, we can exclude a strong inhibition of PP1 via OA under our conditions. The observed effects upon OA treatment are therefore very likely due to inhibition of PP2A or closely related protein phosphatases in intact human platelets.

In early investigations, OA (0.4–1 µM, 10–60 min preincubation, and IC50 ~3.5 µM) inhibited platelet aggregation induced by thrombin, ADP, epinephrine, collagen and TXA_2_ as well as thrombin-induced serotonin release, inositol phosphate formation and thrombin-mediated intracellular Ca^2+^ mobilization [37,38,39]. Furthermore, OA (1 µM, 2–5 min preincubation) inhibited platelet aggregation induced by thrombin and predominantly the second wave of aggregation induced by ADP [79]. Our preliminary data confirmed these earlier studies. 

Furthermore, OA inhibited aggregation of washed human platelets even with much lower OA concentrations (50 nM, 200 nM, 10 min preincubation) in a dose dependent manner in response to thrombin, collagen and TXA_2_ analog. Interestingly, 1 µM OA completely prevented platelet aggregation but not the shape change. We also report that the low OA concentrations (50 nM, 200 nM) increased phosphorylation of several proteins within distinct signaling pathways (Figure 7). Although these functional data clearly support the concept that global inhibition of PP2A by OA results in inhibition of platelet activation it remains to be elucidated, which of the various PP2A subtypes is involved and whether such effects are perhaps PP2A subtype specific. These are important topics for future investigations.

These results show that inhibition of PP2A regulates the phosphorylation and activation of several pathways such as cytoskeletal adaptor proteins (VASP), proteins related to MAPK signalling (MEK, Erk, p38) and PI3K/Akt signalling components. While phosphorylation of VASP, a PKA/PKG substrate, is closely associated with the inhibition of platelet activation [80,81], phosphorylation and activation of MEK, Erk and p38 in platelets is especially important for initiation of platelet activation [51,76,82,83,84] and Akt phosphorylation/activation for amplifying of platelet activation responses [85]. The observed increased phosphorylation of p38 with 200 nM of OA is of considerable interest, since it is well known that there is an extensive cross-talk between p38, other MAP kinases and PP2A [74]. Others showed that PP2A regulates PLA_2_ phosphorylation/activity in human platelets upon platelet activation with thrombin [86] and that p38 is dephosphorylated and therefore inactivated by PP2A [36,85]. 

The observed OA inhibition of thrombin-stimulated platelet aggregation and OA-induced phosphorylation of MAP kinase components and Akt (which all lead to platelet activation) is at first sight a paradox. However, the OA targets PP2A, the MAP kinases and also VASP are components of large modular networks, which may have different functional endpoints. It is well established that PP2A can positively and negatively affect the Erk-MAP kinase pathway in other systems [74].

## 4. Limitations

A S67 ENSA/S62 ARPP19 protein kinase activity has been firmly established for human platelets by our data. However, the precise identity and regulation of this kinase (presumably a MASTL-related protein kinase) remains to be established, which presently is a limitation of our study. Also, while inhibition of platelet PP2A by MASTL-phosphorylated ENSA/ARPP19 has been clearly demonstrated, the precise spectrum of involved PP2A subfamilies is not known yet. Our data also suggest that there are multiple PP2A substrates of different signalling pathways in human platelets. The elucidation of the physiological and pathophysiological function of PP2A in human platelets will require to characterize properties and regulation of the distinct PP2A subtypes and their substrates. However, the similar expression of PP2A subunits in human and murine platelets, as demonstrated here, suggests that murine genetic models with defects or KOs for ENSA/ARPP19 or different PP2A subunits will be important for future studies.

## 5. Conclusions

**Figure 8:**
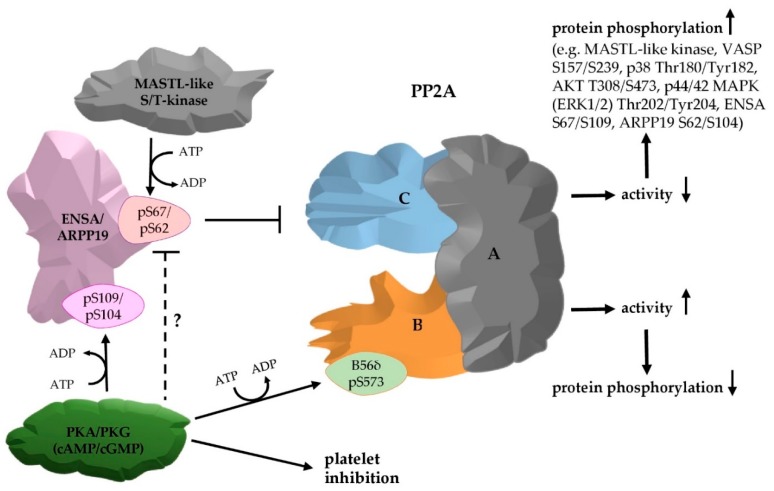
Model of PP2A regulation by S67/S62-phosphorylated ENSA/ARPP19 in human platelets and the impact of PKA/PKG on it. Human platelets contain the two closely related, but distinct proteins ENSA and ARPP19, which are phosphorylated (at S67/62) by MASTL (or a MASTL-like kinase) and then potently inhibit certain PP2A variants, leading to an increase in protein phosphorylation. PP2A enzymes are heterotrimers and may have more than 14 different isozymes in human platelets due to the heterogeneity of the B-subunit. One of them, B56δ, is phosphorylated by PKA, which activates the corresponding heterotrimer, leading to decreased protein phosphorylation. Both platelet PKA and PKG strongly phosphorylate ENSA/ARPP19 at their C-terminus (S109/S104), but the functional consequence of this phosphorylation remains to be elucidated. Platelet PKA/PKG also antagonize MASTL-mediated phosphorylation of ENSA/ARPP19 (S67/S62), but the mechanism needs to be elucidated. PP2A can reduce the phosphorylation of the major PKA subtrate VASP (at S157, S239) and thereby antagonize the PKA pathway. The PP2A family in platelets, which can be potently inhibited by low-dose okadaic acid, dephosphorylates many platelet phosphoproteins as partially shown here. PP2A-mediated dephosphorylation antagonizes the effects of many serine/threonine protein kinases including MAPKs, PKA, PKG and their substrates.

This work shows that the well-established MASTL-ENSA/ARPP19-PP2A pathway, which is essential for the cell cycle control of most dividing eukaryotic cells, is present and regulated in non-dividing, anucleate human platelets (Figure 8).

In our phosphoproteomic studies with intact human platelets, both cAMP-/cGMP-elevating platelet inhibitors strongly induced ENSA/ARPP19 (S109/S104) phosphorylation with concomitant reduced S67/S62 phosphorylation. This suggests an inhibition of MASTL—induced ENSA/ARPP19 phosphorylation by PKA/PKG, similar to an effect of PKA on MAST3 observed in brain [47]. Our data also established that both MASTL- and PKA/PKG-phosphorylated ENSA/ARPP19 are PP2A substrates, whereas only MASTL-phosphorylated ENSA/ARPP19 are PP2A inhibitors. The elucidation of the PKA/PKG phosphorylation effects on ENSA/ARPP19-PP2A function in human platelets may require more detailed kinetic analysis and/or analysis of individual PP2A complexes rather than a mixture of all 14 PP2A forms. 

Generally, it has been shown that dephosphorylation by protein phosphatases such as PP2A could inhibit, stimulate or modify the activity/function of substrate proteins resulting in various cellular effects [26]. In human platelets, we could show that detection of proteins being phosphorylated upon PP2A inhibition is possible. This shifts the equlibrium towards kinase activity/phosphorylation as shown in Figure 7 and Figure 8. It will be of considerable interest to establish a larger spectrum of PP2A substrates in platelets, which is now possible by the methods we have developed. 

Finally, what is the role of a cell cycle checkpoint in the anucleate platelets? We suggest that PP2A checkpoint systems not only control the cell cycle, but also other essential cell functions, especially in platelets. Research efforts with yeast presenting a well-established Greatwall-ENSA-PP2A pathway, very recently emphasized the need to search for novel PP2A substrates and their functions [87,88]. The various PP2A holoenzymes present in human platelets could have overlapping, distinct and also opposing effects on important disease-relevant platelet responses, such as integrin activation, secretion and thrombin generation. Therefore, it will be important to address the substrate and signalling specificity of the various PP2A forms and which of them is inhibited by S67-phosphorylated ENSA, S62-phosphoryated ARPP19 or both.

Due to the enormous PP2A diversity and possibilities of redundant functions, individual PP2A family members and their regulators/inhibitors need to be studied independently. This is now possible using murine and human genetics [72,89,90,91], novel selective PP2A inhibitors [62] and cellular models such as human platelets.

## Figures and Tables

**Table 1:** cAMP-elevating (iloprost) and cGMP-elevating (riociguat, various NO donors) platelet inhibitors regulate VASP and ENSA phosphorylation.

Gene	Uniprot	P-Site	Av. Ratio Iloprost	Av. Ratio DEA-NO	Av. Ratio Riociguat	Av. Ratio SNC	Av. Ratio SNP	Copy Number/Platelet
VASP	P50552	S239	7.17	12.19	9.22	7.67	6.93	44,600
ENSA	O43768	S109	17.97	14.04	16.03	13.88	12.46	7800
ENSA	O43768	S67	0.53	0.82	0.62	0.72	0.82	7800

Washed human platelets were incubated without (control) or with the following test substances : iloprost (5 nM, 2 min); diethylamine NONOate (DEA-NO 5 µM, 2 min.); riociguat (10 µM, 5 min); sodium S-nitrosocysteine (SNC, 5 µM, 2 min) or sodium nitroprusside (SNP, 5 µM, 2 min). The reaction was stopped with 4× lysis buffer, samples were shock frozen in liquid nitrogen and analysed by quantitative phosphoproteomics as described [40]. The change of various phosphosites in response to the test substance compared to control (increase or decrease) is shown as average ratio (av. ratio). The copy number indicates the number of molecules of a given protein per platelet obtained in earlier studies [34].

**Table 2:** Iloprost- and riociguat- stimulated phosphorylation of selected phosphosites of ENSA, ARPP19, PP2A-B56δ (PPP2R5D), and VASP in human platelets.

Gene	Uniprot	Protein Name	Copy Number/Platelet	P-site (1st) (#of Averaged Peptides)	Iloprost (cA)		Riociguat (cG)	
					Av. Ratio	*p*-Value Fraction	Av. Ratio	*p*-Value Fraction
ENSA	O43768	ENSA/ARPP19e	7800	S109 (3)	4.81	100%	3.75	100%
ARPP19	P56211	ARPP19	2500	S104 (1)	3.53	100%	2.18	100%
PPP2R5D	Q14738	PP2A B-subunit B’-δ (B56δ)	1300	S573 (1)	4.52	100%	2.13	100%
VASP	P50552	VASP	44600	S157* (1)	2.03	100%	1.67	100%
VASP	P50552	VASP	44600	S239 (4)	3.92	100%	4.52	100%

Washed human platelets from three healthy donors (three biological replicates) were incubated with buffer (control), iloprost (5 nM, 2 min at 37 °C) or riociguat (10 µM, 5 min at 37 °C). After incubation, samples were stopped by the addition of 4× lysis buffer, shock frozen in liquid nitrogen and analysed by quantitative phosphoproteomics as described [40]. The fold increase of phosphorylation of various phosphosites compared to control is shown as average ratio of all quantified peptides bearing the corresponding site. As a measure of reliability, the ‘*p*-value fraction’ (for each site: ∑ peptides *p* < 0.05/∑ site-bearing peptides) and # of averaged peptides is presented. The change of various phosphosites in response to the test substance compared to control (increase or decrease) is shown as average ratio (av. ratio). The p-value fraction of all sites shown here is 100%, which represents an excellent reliability of these phosphosite measurements. * All trypsin digests except VASP S157 (subtilisin digest).

## Supplementary Materials

The following are available online at https://www.mdpi.com/2073-4409/9/2/472/s1, Table S1. Diversity, copy number and intracellular concentration of serine/threonine protein phosphatases (catalytic subunits) in human platelets. Table S2. Diversity, copy numbers and intracellular concentration of PP2A subunits (catalytic C, scaffolding A, regulatory B) and of ENSA, ARPP19 and VASP in human and murine platelets. Figure S1. Human platelet PP2A B56δ is phosphorylated at S573 by PKA as well as PKG in intact human platelets. Figure S2. Forskolin-effects on the S67-/S109-phosphorylation of endogenous ENSA or transfected FLAG-ENSA in HEK293 cells. Figure S3. Site-specific phosphorylation of HisENSA (wildtype and S67A mutant) by MASTL and PKGIβ in combination. Figure S4. Site-specific phosphorylation of GST-ARPP19 by MASTL kinase, PKGIβ and in combination. Figure S5. Inhibition of the PP2A family in human platelets affects platelet aggregation but not shape change. Figure S6. Inhibition of the PP2A family with 50 nM and 200 nM OA in human platelets affects multiple signaling pathways.
